# Evaluation of Antifungal Properties of Titania P25

**DOI:** 10.3390/mi13111851

**Published:** 2022-10-28

**Authors:** Kunlei Wang, Oliwia Paszkiewicz, Mewin Vincent, Patrycja Henkiel, Damian Kowalski, Ewa Kowalska, Agata Markowska-Szczupak

**Affiliations:** 1 Institute for Catalysis, Hokkaido University, N21, W10, Sapporo 001-0021, Japan; 2 Department of Chemical and Process Engineering, West Pomeranian University of Technology in Szczecin, Piastow 42, 71-065 Szczecin, Poland; 3 Faculty of Chemistry & Biological and Chemical Research Centre, University of Warsaw, Zwirki i Wigury 101, 02-089 Warsaw, Poland

**Keywords:** photocatalysis, P25, enzymatic activity, mold fungi, *Aspergillus fumgiatus*, *Aspergillus niger*

## Abstract

Commercial titania photocatalyst—P25 was chosen for an antifungal property examination due to it exhibiting one of the highest photocatalytic activities among titania photocatalysts. Titania P25 was homogenized first (HomoP25) and then annealed at different temperatures. Additionally, HomoP25 was modified with 0.5 wt% or 2.0 wt% of platinum by a photodeposition method. The obtained samples were characterized by diffuse-reflectance spectroscopy (DRS), X-ray photoabsorption spectroscopy (XPS), X-ray diffraction (XRD) and Raman spectroscopy. Moreover, photocatalytic activity was tested for methanol dehydrogenation under UV/vis irradiation. The spore-destroying effect of photocatalysts was investigated against two mold fungal species, i.e., *Aspergillus fumigatus* and *Aspergillus niger*. Both the mycelium growth and API ZYM (estimation of enzymatic activity) tests were applied for the assessment of antifungal effect. It was found that annealing caused a change of surface properties of the titania samples, i.e., an increase in the noncrystalline part, a growth of particles and enhanced oxygen adsorption on its surface, which resulted in an increase in both the hydrogen evolution rate and the antifungal effect. Titania samples annealed at 300–500 °C were highly active during 60-min UV/vis irradiation, inhibiting the germination of both fungal spores, whereas titania modification with platinum (0.5 and 2.0 wt%) had negligible effect, despite being highly active for hydrogen evolution. The control experiments revealed the lack of titania activity in the dark, as well as high resistance of fungi for applied UV/vis irradiation in the absence of photocatalysts. Moreover, the complete inhibition of 19 hydrolases, secreted by both tested fungi, was noted under UV/vis irradiation on the annealed P25 sample. It is proposed that titania photocatalysts of large particle sizes (>150 nm) and enriched surface with oxygen might efficiently destroy fungal structures under mild irradiation conditions and, thus, be highly promising as covering materials for daily products.

## 1. Introduction

The impact of indoor microclimate on occupant well-being as well as the dangerous influence of mold fungi on air quality are highly important issues for modern human society [1,2,3,4]. The recent two years of the COVID-19 pandemic have revealed other aspects of superinfection associated with common indoor and outdoor molds [5,6]. For example, many people infected with COVID-19 or recovering from COVID-19 have developed Invasive Pulmonary Aspergillosis (IPA) or a similar syndrome associated with severe COVID-19—pulmonary aspergillosis (CAPA). These non-contagious fungal infections are caused by common indoor and outdoor molds from the genera *Aspergillus* sp., with *Aspergillus fumigatus* being the most common pathogen [7]. Other accompanying species include *Aspergillus niger* and *Aspergillus flavus*. These microscopic fungi are widespread in nature and human habitats. The main reservoirs of *Aspergillus sp*. fungi are soil, organic plant debris, food, cereals, nuts, dried fruit and dust (spores make up 5–20% of house dust). An increase of fungal growth occurs at temperature of 20–26 °C and high air humidity (96–98%) [8]. During prolonged human contact with mold, respiratory infections might occur, and other tissues could be attacked. The most frequently described symptoms include fever, cough, hemoptysis, hematemesis, pain breathlessness, increased allergic reactions or tissue necrosis [9]. It should be pointed out that the invasive aspergillosis (IA) is highly lethal. Moreover, the Aspergillus species produces dangerous biochemicals, known as mycotoxins, e.g., aflatoxins (AFTs), ochratoxin A (OTA), patulin (PAT), citrinin (CIT), aflatrem (AT), secalonic acids (SA), cyclopiazonic acid (CPA), terrein (TR), sterigmatocystin (ST) and gliotoxin (GT), etc. Most of them are thermostable and have mutagenic, genotoxic, hepatotoxic, teratogenic, and carcinogenic effects at nanogram concentrations [10]. Infections caused by Aspergilli are difficult to treat due to resistance to azole drugs (e.g., voriconazole, isavuconazole, posaconazole, etc.) [9], as a direct consequence of soil, wastewater and sewage sludge contamination with azoles used as biocides [11]. Accordingly, it is extremely important to find effective methods to inhibit the growth of these fungi. In order to solve this problem, various efforts have been undertaken, including photocatalysis based on titanium(IV) oxide (titania).

Photocatalysis is a process in which light energy (UV, vis or IR) is used to initiate redox reaction on the surface of photocatalysts [12]. In the case of titania (the most famous, and widely used photocatalysts), UV light must be applied because of its wide bandgap (ca. 2.9–3.2 eV, depending on the type of its polymorphs and purity). After UV absorption, electrons are photoexcited from the valence band (VB) to the conduction band (CB). Formed charge carriers (electrons in CB and holes in VB) might either migrate to the photocatalyst surface or recombine (in the bulk or on the surface [13]). Of course, the recombination is unwanted and results in lower quantum yields of photochemical reaction than theoretical 100%, whereas the charge carriers on the surface might initiate various redox reactions, including the formation of reactive oxygen species (ROS), e.g., famous hydroxyl radicals, or direct reactions with chemical and microbiological matters. To hinder charge carriers’ recombination and to extend photoactivity towards vis range of solar spectrum, titania (and other wide-bandgap semiconductors) has been modified, doped, nano-structured and combined with other materials (heterojunction, Z-scheme, etc.) [14,15,16,17,18,19]. Among various modifiers, noble metals (NMs), especially platinum, have been broadly investigated since NMs might scavenge photogenerated electrons (hindering their recombination with holes [20,21,22,23], and, thus, increasing the activity under UV) and activate titania towards vis response via plasmonic photocatalysis [24,25,26].

Nowadays, the “green” technology based on titania photocatalysis to control fungal growth has been mainly applied in the building industry. The available products with antifungal properties include indoor and outdoor paintings, cements and coatings. The main producers are big international companies, such as, Intalcementi HeidelbergCement Group, (Heidelberg, Germany), R&G Global Engineering (Barnsley, UK), Sigma Coatings (Houston, TX, US), Rokospol a.s., Sto SE & Co. KGaA (Stühlingen, Germany), Okitsumo Incorporated (Shibade, Japan), EcogreenPlus (Anaheim, CA, USA), Brillux (Muenster, Germany), Pacific Paints (Manila, Philipines), Green Millennium (Walnut, CA, USA), SolGelWay (Sceaux, France), Pureti (Cincinnati, OH, USA), TitanPE Technologies Inc. and Selena S.A., (Wroclaw, Poland), Protectam, Sp.z.o.o (Klodzko, Poland). However, there is some disagreement between laboratory results and manufacturers’ declarations. According to previous research [27,28,29,30], fungi are highly resistant to photocatalytic treatment, and, thus, it is very hard to remove them from the surface of building materials. Moreover, there is a lack of one standard for determining antifungal activity. At present, research teams use different methods to estimate antifungal efficiency. Most approaches are based on the estimation of the number of fungal spores or growth inhibition zones in comparison with negative control (without photocatalyst) or positive control (without light as activator of photocatalytic process) [3,27,28,31,32]. Since almost all-important factors, such as microbial species/strains, irradiation conditions (light intensity, wavelength range, time of exposure, illumination distance), temperature, humidity and photocatalyst (dose and properties), are varied, no direct comparison is possible. In addition, the microorganisms selected for the tests do not occur in their real environment, which is a huge obstacle for the exploitation of experimental results in the development of photocatalytic technology.

Accordingly, the main goal of this study is to propose a better method for testing of antifungal activity, i.e., the method based on the estimation of enzymatic activity. The huge group of hydrolases (EC 3) has been investigated. According to the definition, these enzymes catalyze the hydrolysis of chemical bonds in biomolecules, such as lipids, proteins and carbohydrates. The COVID-19 accompanying species, i.e., *Aspergillus niger* and *Aspergillus flavus*, and the famous titania P25-based photocatalysts (with one of the highest photocatalytic activities in different redox reactions [33,34,35,36]) both pristine and modified, i.e., thermally treated (to change particle size) and platinized, have been selected for the tests. The photocatalytic activity for hydrogen evolution reaction has also been examined to compare photo-reduction activity with antifungal performance.

## 2. Materials and Methods

### 2.1. Preparation of Photocatalysts

At first, the original P25 (Evonik/Nippon Aerosil, Tokyo, Japan) was homogenized by suspending it in water, mixing for 24 h, and then freeze drying, as reported previously [33,34]. The sample was named as HomoP25 and was used for the preparation of other photocatalysts. Three samples were prepared by simple annealing of HomoP25 at different temperatures, i.e., 200, 300 and 500 °C, and named accordingly as HomoP25-200, HomoP25-300 and HomoP25-500. The annealing was carried out under air atmosphere in a rotary furnace. The time needed to reach the annealed temperature was fixed to 0.5 h. The samples were kept in the oven at set temperature for 2.5 h. They were then left to cool down to room temperature.

Additionally, HomoP25 was also modified with platinum by the photodeposition method. In brief, HomoP25 was suspended in methanol solution (50 vol%), to which aqueous solution of H_2_PtCl_4_ (Wako Chemicals, Tokyo, Japan) was added, and then air was removed from the tube by argon (Ar) bubbling. Photodepositiona was performed under UV/vis irradiation using a high-pressure mercury lamp (wavelength < 290 nm) for 1 h. Two different samples were prepared, i.e., 0.5Pt-HomoP25 and 2.0Pt-HomoP25, depending on the content of deposited platinum (to the weight of titania), i.e., 0.5 and 2.0 wt%, respectively.

### 2.2. Characterization of Photocatalysts

All samples were characterized by various methods and techniques, as follows. Crystalline composition was investigated by the X-ray diffraction (XRD) on a Rigaku Intelligent X-ray diffraction system SmartLab equipped with: a sealed tube X-ray generator (a copper target; operated at 40 kV and 30 mA), a D/teX high-speed position sensitive detector system and an ASC-10 automatic sample changer. The crystallinity was estimated by the internal standard method, with nickel oxide (20.0 wt%; Wako Chemicals, Tokyo, Japan) as a standard of high crystallinity. The absorption properties were measured by diffuse reflectance spectroscopy (DRS), carried out on a spectrophotometer (Jasco V-670), equipped with a PIN-757 integrating sphere. The baseline was recorded using BaSO_4_ (Wako Chemicals, Tokyo, Japan) as a reference. The chemical composition of the surface (content and chemical state of elements, i.e., titanium, oxygen and platinum) was determined by X-ray photoelectron spectroscopy (XPS; JEOL JPC-9010MC with MgKα X-ray, JEOL, LTD., Tokyo, Japan); each element was scanned 100 times. Particle size was estimated using a laser diffraction particle analyzer (SALD-7000 Shimadzu). Surface chemical properties were analyzed with a micro Raman spectrometer (DXR3 Thermo Fisher Scientific, Waltham, USA) fitted with a 532-nm Nd:YAG laser. Measurements were carried out at 2% laser power to prevent sample degradation. Additionally, a scanning transmission electron microscopy (STEM) was used for the observations of the titania surface (STEM, Hitachi HD2000, Tokyo, Japan).

### 2.3. The Photocatalytic Activity for Hydrogen Evolution under UV/vis Irradiation

The photocatalytic activity of samples was tested under UV/vis irradiation in dehydrogenation of methanol with platinum (Pt) in-situ photodeposited on titania. Photocatalytic activity was examined by measuring the amount of evolved H_2_ from continuously stirred suspensions of a sample (50 mg) in an aqueous solution of methanol (5.0 mL, 50 vol%). During the irradiation, a portion (0.2 mL) of the gas phase of reaction mixture was withdrawn with a syringe and subjected to gas chromatographic analysis (TCD-GC, Shimadzu GC-8A-IT, Tokyo, Japan).

### 2.4. Anifungal Tests

The antifungal tests were conducted for *Aspergillus* strains *A. fumigatus* (ZUT 1) and *A. niger* (ZUT 2) from the collection of the Department of Chemical and Process Engineering. Both strains were isolated from the air of the fitness club there by the sedimentation method. The species identification was carried on the basis of analysis of morphological features and biochemical analysis.

#### 2.4.1. Mycelial Growth Test

Ten-day old fungal cultures grown on Malt Extract Agar (MEA, Merck, Darmstadt, Germany) slants at 25 °C were used for the preparation of spore suspensions. A total of 5 mL of sterile physiological saline buffer (0.85% NaCl) was added to each slant. Then, the slants were vigorously shaken by a vortex for 3 min. The working solution of fungal spores was prepared at the concentration of 7.6 log CFU/mL. Then, a 0.2 g of sample was dispersed in 10.0 mL of the working solution in two test tubes. One test tube was incubated in the dark, while another was exposed to artificial solar light (UV/vis; ULTRA-VITALUX 230 V E27/ES, OSRAM 300 W, Munich, Germany). The distance from the lamp was set at ca. 10 cm. The temperature was kept constant (25 °C) during the whole incubation period. The samples were withdrawn from the tubes for analysis at 0, 60, 120 and 180 min, diluted and inoculated on a MEA. Then, the plates were incubated at 25 °C. The visible colonies were counted after 3 days (72 h). The experiment was verified three times. The statistical significance of the differences in daily growth rates was evaluated with a one-way ANOVA test at *p* ≤ 0.05.

#### 2.4.2. Enzymatic Activity

The standard fungal media Malt Extract Agar (MEA; Merck, Darmstadt, Germany) supplemented with HomoP25-300 (20 g/L) was used for enzymatic activity testing. The media free of titania served as a control. The temperature was kept at 25 °C during the whole incubation period (72 h). After the incubation, half of the Peri dishes (containing cultures of fungi) were incubated in the dark, while the others were exposed to artificial solar light (UV/vis; the same lamp and irradiation conditions as those described in 2.4.1) for 60 min. The spores from the center of the mycelium were collected, and the spore suspensions (approx. density of 7.6 log CFU/mL) were prepared. Then, 0.02 mL of prepared fungal spore suspension was dropped into a stripe of API-ZYM test. This system consists of a plastic gallery containing 20 cupules. Cupule No. 1 contains no substrate and acts as a negative control, whereas the 19 others have substrates and buffer impregnated into inert supportive fabric. The hydrolytic enzymes and their substrates assayed using the commercial API ZYM test (BIOMÈRIEUX, Craponne, France) are listed in Table 1. Tests were conducted according to the procedure of the manufacturer.

All strips were incubated aerobically at 37 °C for 4 h. After incubation, 0.04 mL of the API reagents A and B were added to each cupule and exposed for 30 s to a strong light source (1000-Watt lamp). The resulting colors were recorded, being classified by a semiquantitative notation (0 to 5) using a color code supplied by the manufacturer. The color intensity of enzymatic reactions represents the enzymatic activity of each sample and the quantity of the hydrolyzed substrate. For example, dark pink (“5”), pink (“3”), light pink (“1”) and white (“0”) correspond to strong activity (>40 nM), moderate activity (20 nM), low activity (5 nM) and no activity (0 nmol).

## 3. Results

### 3.1. Characterization of Photocatalysts

**Figure 1:**
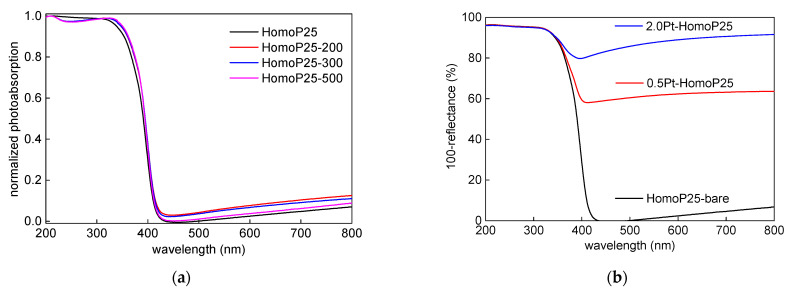
The photoabsorption properties (DRS) of: (**a**) pristine; and (**b**) Pt-modified samples.

**Figure 2:**
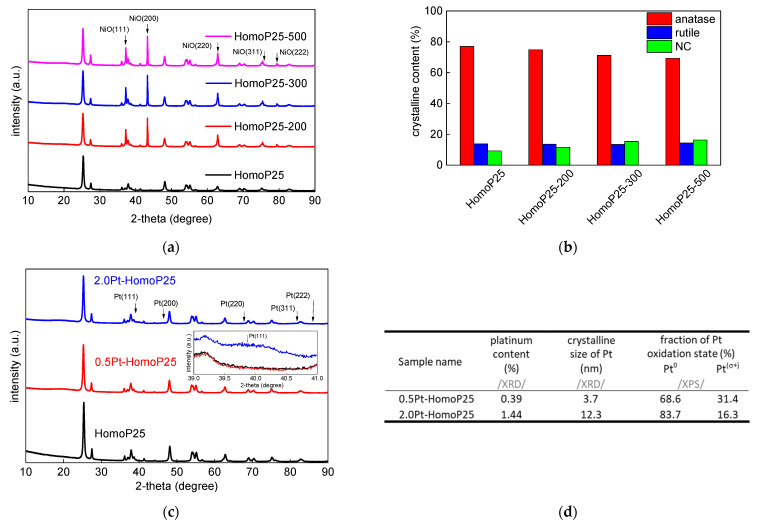
(**a**–**c**) Crystalline properties of pristine and Pt-modified P25 samples: (**a**) XRD patterns of P25 samples; (**b**) Distribution of phases in P25 samples; (**c**) XRD patterns of Pt-modified HomoP25 samples (magnification of Pt(111) region shown in inset); (**d**) The properties of platinum deposits.

The photoabsorption properties of pristine titania samples are shown in Figure 1a. Titania P25 is composed of two crystalline forms of titania (anatase and rutile) and a noncrystalline (NC) part (mainly amorphous titania) [34,35,37]. Accordingly, photoabsorption edge of all P25-based samples corresponds to the rutile phase (narrower bandgap of ca. 3.0 eV). The annealing might result in both an increase in the particle size (resulting from the particle aggregation) and the phase conversion, i.e., amorphous to anatase, and anatase to rutile. The slight increase in “photoabsorption” at longer wavelengths indicates an increase in particle size and, thus, enhanced scattering. Indeed, both the particle size increase and the change in crystalline properties have been confirmed, as shown in Figure 2a–c and Table 2. After modification with platinum, significant vis response is observed, depending on the content of platinum (Figure 1b), as commonly presented [23,34,38,39,40]). Driessen et al. reported that photoabsorption properties of Pt-modified titania, predicted by the Maxwell–Garnett theory, increases with an increase in the volume fraction of platinum [41].

The presence of anatase and rutile is clearly observed in XRD patterns of all samples, together with a standard (NiO), used for the determination of NC content, as shown in Figure 2a. With an increase in annealing temperture, the crystalline properties have slightly changed, as clearly shown in Table 2 and Figure 2b. As expected, the desposition of platinum on the surface of titania has not changed its properties (Figure 2c). The small platinum peaks could be observed in 2.0Pt-HomoP25 at ca. 39.8°, 46.2°, 67.5°, 81.3° and 85.7°, corresponding to (110), (200), (220), (311) and (322) planes, respectively. The properties of platinum deposits, estimated from XRD and XPS analyses, are summarized in Figure 2d. Since platinum photodeposition on the titania surface is usually very fast (reaction completion within several minutes), it commonly results in the aggregation of formed platinum nanoparticles (NPs) [34,42,43]. Accordingly, larger content of platinum used for photodeposition causes the formation of its larger NPs.

**Figure 3:**
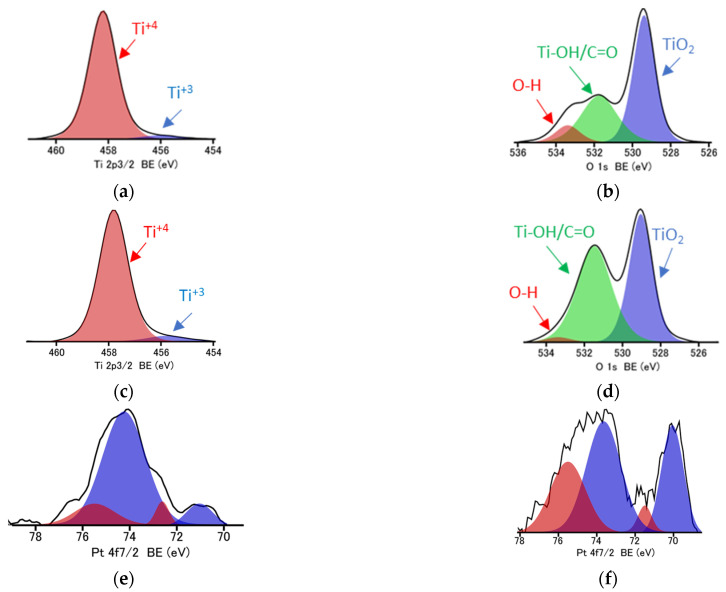
XPS data for HomoP25 (**a**,**b**), HomoP25-500 (**c**,**d**), 0.5Pt-HomoP25 (**e**) and 2.0Pt-HomoP25 (**f**) after deconvolution of Ti 2p_3/2_ (**a**,**c**), O 1 s (**b**,**d**) and Pt 4f_7/2_ (**e**,**f**) peaks.

Then, surface properties of samples were investigated by XPS analysis, and obtained data are shown in Table 3 and Figure 2d and Figure 3. Interestingly, the annealing causes the significant enrichment of the photocatalyst surface with oxygen, changing the O/Ti ratio from 2.6 to 2.9, 2.9 and 3.7 when treated at 200 °C, 300 °C and 500 °C, respectively. Accordingly, it might be concluded that an increase in the content of the NC phase after annealing could be caused by enhanced adsorption of oxygen-based compounds on the titania surface, e.g., water, air and carbon dioxide. Indeed, the deconvolution of oxygen peak confirms that the content of oxygen bound to the surface (not lattice TiO_2_) has increased significantly, i.e., from 34.6% to 51.7% when HomoP25 sample was annealed at 500 °C, as shown in Figure 3. Moreover, the slight increase in the content of reduced titanium (Ti^3+^) is observed in all annealed samples. Lira et al. have proposed that an excess charge (Ti^3+^) could be withdrawn from the titania lattice to oxygen species on the surface and Ti interstitials could react with O adatoms upon heating [44]. In the case of platinum, despite the fact that zero-valent platinum (blue parts in Figure 3e–f) is formed during photodeposition (strong reductive conditions), its surface oxidation results in the co-participation of both positively (red parts in Figure 3e–f) and zero-valent charged platinum (metallic core and oxidized shell), as already reported for other Pt/TiO_2_ samples [45].

**Figure 4:**
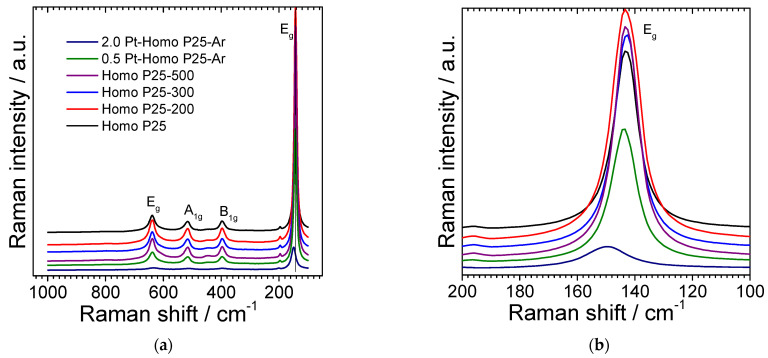
Raman spectra of all samples: (**a**) broad-wavenumber range; (**b**) narrow-wavenumber range.

Next, Raman spectra were recorded for all samples (Figure 4). The typical features of the anatase could be clearly observed with the bands at 144 (E_g_), 399 (B1_g_), 513 (A1_g_) and 639 cm^−1^ (E_g_). The shift of the main vibrational mode peak to higher wavenumber after titania modification with platinum is typical and commonly reported for titania samples modified with noble metals (Au, Pt, Pd) [46,47,48,49,50]. It was proposed that broadening and blue shift of peaks could be caused by an increase in the content of defects (e.g., oxygen vacancies, causing the titania lattice to shrink and, thus, shortening the length of the Ti-O bond) [46,47] and/or substitution of titanium (Ti^4+^) by metal (e.g., Cu, Fe and Mn [50]). However, it was also proposed that platinum could repair the rupture of the Ti–O bond and, thus, reduce the blue shift of Raman peak (though in all Pt-modified samples, the blue shift was observed in comparison to the original titania) [51]. Interestingly, there are also contrary reports showing the red shift of the titania peak after modification with platinum resulting from the growth of particle size (during synthesis) [52]. Nevertheless, the shift of the main peak is commonly reported for titania modified with noble metals, and thus, indirectly confirms the metal presence on the surface of the photocatalysts. Moreover, the significant drop in the relative band intensities also denotes the surface modification with platinum. The noble metal atoms typically disrupt the crystal symmetry of the titania lattice, and thus, significantly impact the lattice vibrational modes of the host.

**Figure 5:**
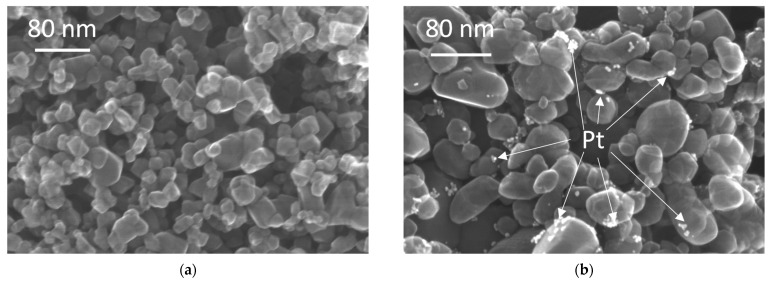
STEM images of photocatalysts: (**a**) HomoP25; and (**b**) 2.0Pt-HomoP25.

Additionally, STEM observations have been conducted, and obtained data are shown in Figure 5. HomoP25 is composed of titania NPs of different shapes and sizes, ranging from ca. 10 nm to ca. 200 nm. It has been estimated that larger and smaller NPs are mainly composed of rutile and anatase, respectively [34]. In the case of Pt-modified samples, aggregated platinum NPs are nonuniformly deposited on the surface of titania, as clearly shown in Figure 5b.

### 3.2. Hydrogen Evolution under UV/vis Irradiation

**Figure 6:**
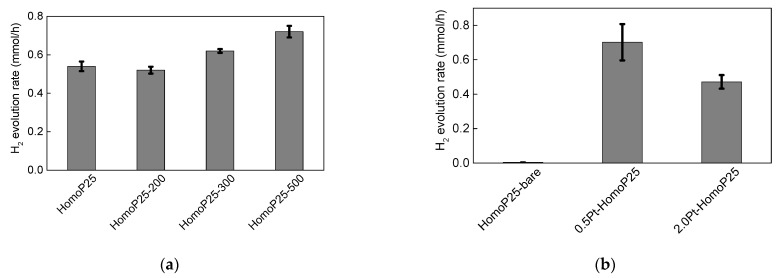
Photocatalytic activity data for methanol dehydrogenation on: (**a**) Pristine titania (in-situ Pt deposition); (**b**) Pristine and Pt-modified (ex-situ Pt deposition, i.e., pre-deposited before activity testing) HomoP25 titania.

The photocatalytic activity of all samples was investigated for methanol dehydrogenation reaction. Since titania is hardly active for gas evolution reactions (both hydrogen and oxygen), because of high overpotential, a metallic co-catalyst is commonly used. Here, platinum was in-situ deposited during activity testing on the surface of pristine titania samples, and obtained results are shown in Figure 6a. Although, similar activities were obtained for HomoP25 and HomoP25-200, the higher annealing temperatures used for the samples’ preparation cause an acceleration of photocatalytic activity, which could be caused by the formation of larger particles or slight surface modification (as observed by XRD and XPS data—an increase in NC content and enhanced oxygen adsorption, respectively). Prieto-Mahaney et al. compared the properties and photocatalytic activities of 35 different titania photocatalysts, finding that an increase in particle size is beneficial for hydrogen evolution [36]. The enrichment of titania surface with oxygen could also be profitable for facilitating platinum adsorption, e.g., platinum encapsulation by an amorphous TiO_x_ cover layer in an oxidative atmosphere [53]. In Figure 6b, the activities of Pt/TiO_2_ samples were compared to the activity of original titania (without in-situ deposition of Pt). Indeed, bare titania is almost inactive for hydrogen evolution, and metallic co-catalyst has increased titania performance by more than one order in magnitude (ca. 20 and 13 times for 0.5Pt-HomoP25 and 2.0Pt-HomoP25, respectively). Moreover, a lower content of platinum shows to be better, which is directly connected with the “shielding” effect, i.e., a decrease in photoabsorption by titania (competing with platinum for photons), as previously reported for optimized conditions of hydrogen evolution on platinized titania [34,43].

### 3.3. Anifungal Properties

**Figure 7:**
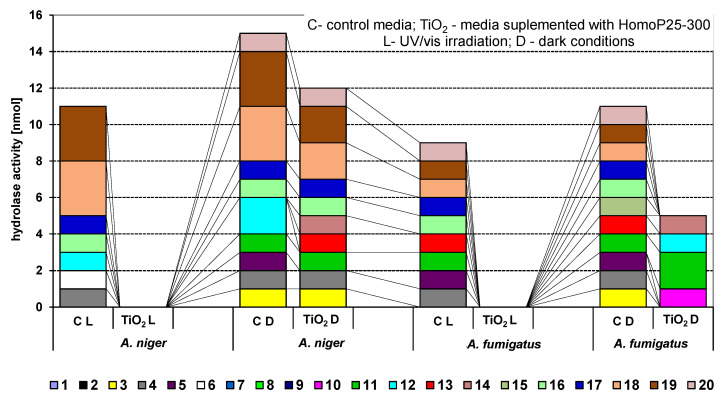
The activity of 19 hydrolases secreted by *A. niger* and *A. fumigatus* fungi growing on control media and supplemented with 20 g/L HomoP25-300 under UV/vis irradiation and in the dark.

The antifungal activities were tested for two *Aspergillus* species known as an important cause of CAPA: *A. niger* and *A. fumigatus.* These were isolated from the air in a fitness club. In dark conditions, the reduction of fungal spores was not observed after 180 min of exposition to all tested photocatalysts. The same results were obtained under UV/vis irradiation but without photocatalysts. The complete inhibition of *A. niger* and *A. fumgiatus* growth occurred after exposition to HomoP25-500 activated under UV/vis for 60 min (Table 4). Moreover, the growth inhibition of the *A. niger* species caused by HomoP25-300 was also very high, and complete inhibition was achieved for both fungi after 120 min of irradiation (Table 3). Unexpectedly, modification with platinum hardly improved the antifungal performance against both *Aspergilli* species in contrast to highly efficient gold-modified titania [54]. For further study (API ZYM test), HomoP25-300 was selected. The results of the enzymatic activity (API ZYM test) for 19 different hydrolases (Table 1) are presented in Figure 7.

It was found that some substrates were never metabolized, e.g., 7, 8 and 9. Most of the enzymes produced by *A. fumgiatus* and *A. niger* were characterized by low activity (“1”) in control media (without TiO_2_) under dark conditions. The only two exceptions were Naphthol-AS-BI-phosphohydrolase (no. 12) and N-acetyl-β-glucosaminidase (no. 18) secreted by *A. niger*, with enzyme activity of “2” and “3”, respectively. Generally, UV/vis had no effect on enzymatic activity in control media. On the contrary, in the presence of titania, the inhibitory effect was significant, especially under irradiation. Under dark conditions, the addition of titania to the media inhibited completely the enzymatic activity of lipase C14 (no. 5) and Naphthol-AS-BI-phosphohydrolase (no. 12) secreted by *A. niger* and of alkaline phosphatase (no. 2), esterase C4 (no. 3), esterase lipase C8 (no. 4) and lipase C14 (no. 5) secreted by *A. fumgiatus.* Interestingly, some enzymes were stimulated by the addition of titania, such as α-chymotrypsin (no. 10), acid phosphatase (no. 11), naphthol-AS-BI-phosphohydrolase (no. 12), α-galactosidase (no. 13), β-galactosidase (no. 14) and α-mannosidase (no. 19). Under UV/vis irradiation, complete inhibition of all enzymes, produced by both types of fungi, was achieved (Figure 7).

## 4. Discussion and Conclusions

Both tested fungi (*Aspergiilus niger* and *Aspergillus fumigatus*) belong to the genus Aspergilli, which consists of more than a hundred opportunistic mold species. They can be found in different climatic conditions, having a high nutritional versatility, which enables them to grow on a large variety of construction materials. Consequently, the exposure to Aspergilli species might be common in the indoor environment (e.g., at home, at school and at the workplace). Most Aspergilli species cause various diseases, including allergic responses, rhinitis bronchopulmonary aspergillosis (e.g., CAPA) and severe asthma with fungal sensitization. The Aspergillus conidia (spores) as well mycelium or hyphal fragments might be produced in a huge number, and they are easily disseminated in air, especially in places with poor ventilation [55]. Additionally, a byproduct of fungal mycelial structures or spores are mycotoxin—a toxic secondary metabolite. All of them are potentially hazardous for humans and animals [56]. Because of health-related implications, the removal or/and neutralization of fungal spores is very important. This study has focused on the photocatalytic inhibition of fungal germination by using six titania photocatalysts, activated under UV/vis irradiation. The inactivation of the spores produced by two *Aspergilli* species was investigated according to the agar plate method and the API ZYM test. It was found that the thermal treatment of titania was more prominent than modification with platinum for enhanced antifungal properties. The complete inactivation of germination (with 7.6 log CFU/mL initial fungal spore concentration) was achieved within 60-min irradiation for HomoP25-500. Additionally, under the same conditions, high fungicidal response was achieved for HomoP25-300, i.e., for *A. fumigatus* spores the lethal conditions were obtained within 60 min, whereas, for *A. niger,* longer irradiation (120 min) was necessary. The inactivation effect was not observed in dark conditions. Similar data (but under dark conditions) have recently been obtained by Noman et al. [57]. Bio-nanoparticles of silver (synthesized as secondary metabolic products of *Penicillium pedernalens* 604 EANe) were efficient to inactivate completely (6 log reduction with 99.9999% efficiency) *A. aculeatus* during 110 min of treatment. However, a longer time period (10 min) was necessary to insure the irreversible inactivation of the fungal spores [57].

Interestingly, the lack of activity of platinum could be caused by high adsorption capacity for Pt by Aspergillus fungi, which has even been proposed as a method for the removal of metals (including heavy metals) from the environment [58]. To our knowledge, there are no studies on the application of Pt-modified titania for the removal of mold fungi. In contrast, nitrogen-, fluorine-, gold- and silver-modified titania samples exhibit superior antifungal activity against *Fusarium oxysporum* and *Penicillium chrysogenum* [27,54,59]. Additionally, the synergistic effect between silver and cupric oxide, deposited on the surface of titania, was reported for *Penicillium chrysogenum* and *Aspergillus melleus* [60]. However, the antifungal effect was stronger in the dark than under irradiation and only significant for specific ratio between components (i.e., 1:3 of Ag to CuO).

For enzyme activity tests (API ZYM), the spores collected from three-day fungal cultures growing on MEA media supplemented with 20 g/L HomoP25-300 were used. Before the test, 60-min activation of photocatalyst was done to simulate the growth conditions on the surface of building materials, additionally allowing them to obtain a spore suspension (necessary for the test) without titania. It is noteworthy that the fungal growth was much slower for MEA media with titania. The complete activation of 19 hydrolases occurred after 60 min of activation of HomoP25-300 under UV/vis. It was observed that even conidia germination was highly disturbed. It is well known that many different enzymes are involved in mycotoxin production or conidia formation/germination. In particular, hydrolases present a biochemical meaning to fungal growth and development because of the acquisition of nutrients from the surrounding environment [61]. Although the crystalline and nanosized particles are expected to show higher activity, titania characterized by larger particles (HomoP25-500) possesses stronger antifungal effect, which might be explained by depletion of organic nutrients from the media, resulting from the adsorption of titania on the microorganisms’ surface. Taking into account the significance of hydrolases (one of the biggest enzyme classes), it is highly probable that the inactivation of enzymes leads to the fungal death.

A huge number of physical, chemical and biological strategies for the elimination of mold fungi (or formation of mycotoxin) have already been reported in the literature [62,63,64,65,66,67]. Based on the present results, it is clear that photocatalysis is an extremely effective and environmentally friendly (without consumables and harmful residues) method for fungal inactivation. Moreover, the enzymatic activity test has proven to be recommended for fast and relatively cheap evaluation of the antimicrobial (particularly antifungal) activity of photocatalysts. However, it should be remembered that further studies are necessary to validate if this method could be used for building products (e.g., paints, coatings, wallpapers), filters and other materials containing photocatalytic active titania.

## Figures and Tables

**Table 1:** Tested hydrolase and their substrates.

No	Enzyme	Substrate
1.	Control	
2.	Alkaline phosphatase	2-naphtyl phosphate
3.	Esterase (C4)	2-naphtyl butyrate
4.	Esterase Lipase (C8)	2-naphtyl caprylate
5.	Lipase (C14)	2-naphtyl myristate
6.	Leucine arylamidase	L-leucyl-2-naphthylamide
7.	Valine arylamidase	L-valyl-2-naphthylamide
8.	Cystine arylamidase	L-valyl-2-naphthylamide
9.	Trypsin	N-benzoyl-DL-arginine-2-naphtylamide
10.	α-chymotrypsin	N-glutatyl-phenylalanine-2-naphthylamide
11.	Acid phosphatase	2-naphthyl phosphate
12.	Naphthol-AS-BI-phosphohydrolase	Naphthol-AS-BI-phosphate
13.	α-galactosidase	6-Br-2-naphthyl-αD-galactopyranoside
14.	β-galactosidase	2-naphthyl-βD-galactopyranoside
15.	β-glucuronidase	Naphthol-AS-BI-βD-glucuronide
16.	α-glucosidase	2-naphthyl-αD-glucopyranoside
17.	β-glucosidase	6-Br-2-naphthyl- βD-glucopyranoside
18.	N-acetyl-β-glucosaminidase	1-naphthyl-N-acetyl-βD-glucosamide
19.	α-mannosidase	6-Br-2-naphthyl- αD-mannopyranoside
20.	α-fucosidase	2-naphthyl-αL-fucopyranoside

**Table 2:** The properties of pristine titania photocatalysts.

Sample Name	Crystalline Composition (%)			Particle Size (nm)
	Anatase	Rutile	NC	
HomoP25	77.0	13.8	9.2	119.1
HomoP25-200	74.8	13.6	11.6	141.6
HomoP25-300	71.2	13.4	15.4	187.8
HomoP25-500	69.3	14.4	16.3	521.9

NC—non-crystalline phase.

**Table 3:** The surface properties of pristine titania photocatalysts.

Sample Name	Oxygen (1 s)			Titanium (2p_3/2_)		O/Ti Molar Ratio
	O-H	Ti-OH/C=O	TiO_2_	Ti^4+^	Ti^3+^	
HomoP25	8.6	34.6	56.8	96.4	3.6	2.6
HomoP25-200	14.1	33.1	52.8	93.4	6.6	2.9
HomoP25-300	10.4	37.8	51.8	95.8	4.2	2.9
HomoP25-500	2.1	51.7	46.2	95.1	4.9	3.7

NC—non-crystalline phase.

**Table 4:** The antifungal activity of photocatalyst under UV/vis (no activity in the dark).

Photocatalysts Name	Negative Control	HomoP25	HomoP25-200	HomoP25-300	HomoP25-500	0.5Pt-HomoP25	2.0Pt-HomoP25	Negative Control	HomoP25	HomoP25-200	HomoP25-300	HomoP25-500	0.5Pt-HomoP25	2.0Pt-HomoP25	Negative Control	HomoP25	HomoP25-200	HomoP25-300	HomoP25-500	0.5Pt-HomoP25	2.0Pt-HomoP25
irradiation [min]	60							120							180						
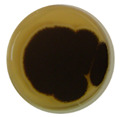 *A. niger* [logCFU/mL]	7.6	7.1	7.0	0.0	0.0	7.3	7.2	7.6	6.9	6.7	0.0	0.0	7.1	7.1	7.5	6.6	6.5	0.0	0.0	6.8	6.4
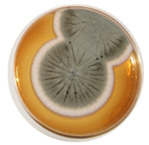 *A. fumigatus* [logCFU/mL]	7.6	7.6	6.8	5.1	0.0	7.4	6.2	7.6	7.0	6.2	0.0	0.0	7.2	6.1	7.6	7.0	6.1	0.0	0.0	6.9	5.8

## Data Availability

The data presented in this study are available on request from corresponding author (E.K.).

## References

[1] Gonzalez-Martin J., Kraakman N.J.R., Perez C., Lebrero R., Munoz R. (2021). A state-of-the-art review on indoor air pollution and strategies for indoor air pollution control. Chemosphere.

[2] Kanchongkittiphon W., Mendell M.J., Gaffin J.M., Wang G., Phipatanakul W. (2015). Indoor Environmental Exposures and Exacerbation of Asthma: An Update to the 2000 Review by the Institute of Medicine. Environ. Health Perspect..

[3] Markowska-Szczupak A., Wang K.L., Rokicka P., Endo M., Wei Z.S., Ohtani B., Morawski A.W., Kowalska E. (2015). The effect of anatase and rutile crystallites isolated from titania P25 photocatalyst on growth of selected mould fungi. J. Photoch. Photobio. B.

[4] Markowska-Szczupak A., Janda K., Wang K.L., Morawski A.W., Kowalska E. (2015). Effect of Water Activity and Titania P25 Photocatalyst on Inactivation of Pathogenic Fungi—Contribution to the Protection of Public Health. Cent. Eur. J. Publ. Health.

[5] Lai C.C., Yu W.L. (2021). COVID-19 associated with pulmonary aspergillosis: A literature review. J. Microbiol. Immunol. Infect..

[6] Salas B., McCullagh I., Cranfield K., Fagan C., Geering A., Robb A. (2022). COVID-19-Associated Pulmonary Aspergillosis: A Year-Long Retrospective Case Series. COVID.

[7] Dimopoulos G., Almyroudi M.-P., Myrianthefs P., Rello J. (2021). COVID-19-Associated Pulmonary Aspergillosis (CAPA). J. Intensive Care Med..

[8] Pratiwi C., Rahayu W., Lioe H., Herawati D., Broto W., Ambarwati S. (2015). The effect of temperature and relative humidity for Aspergillus flavus BIO 2237 growth and aflatoxin production on soybeans. Int. Food Res. J..

[9] Lal P.M., Arif A., Mohan A., Rackimuthu S., Hasan M.M., Islam Z., Uday U., Wara U.U., Shaikh M.T.A., Essar M.Y. (2022). COVID-19 associated pulmonary aspergillosis (CAPA): An added potential burden on India’s pre-existing fungal superinfection. Clin. Epidemiol. Glob. Health.

[10] Navale V., Vamkudoth K.R., Ajmera S., Dhuri V. (2021). Aspergillus derived mycotoxins in food and the environment: Prevalence, detection, and toxicity. Toxicol. Rep..

[11] Burks C., Darby A., Gómez Londoño L., Momany M., Brewer M.T. (2021). Azole-resistant Aspergillus fumigatus in the environment: Identifying key reservoirs and hotspots of antifungal resistance. PLoS Pathog..

[12] Ohtani B. (2014). Revisiting the fundamental physical chemistry in heterogeneous photocatalysis: Its thermodynamics and kinetics. Phys. Chem. Chem. Phys..

[13] Herrmann J.-M., Disdier J., Pichat P., Malato S., Blanco J. (1998). TiO_2_-based solar photocatalytic detoxification of water containing organic pollutants. Case studies of 2,4-dichlorophenoxyaceticacid (2,4-D) and of benzofuran. Appl. Catal. B Environ..

[14] Zaleska A. (2008). Doped-TiO_2_: A review. Rec. Patent. Eng..

[15] Mitoraj D., Kisch H. (2008). The nature of nitrogen-modified titanium dioxide photocatalysts active in visible light. Angew. Chem. Int. Ed..

[16] Mitoraj D., Janczyk A., Strus M., Kisch H., Stochel G., Heczko P.B., Macyk W. (2007). Visible light inactivation of bacteria and fungi by modified titanium dioxide. Photochem. Photobiol. Sci..

[17] Wang K., Bielan Z., Endo-Kimura M., Janczarek M., Zhang D., Kowalski D., Zielińska-Jurek A., Markowska-Szczupak A., Ohtani B., Kowalska E. (2021). On the mechanism of photocatalytic reactions on Cu_x_O@TiO_2_ core–shell photocatalysts. J. Mat. Chem. A.

[18] Wang K.L., Janczarek M., Wei Z.S., Raja-Mogan T., Endo-Kimura M., Khedr T.M., Ohtani B., Kowalska E. (2019). Morphology- and crystalline composition-governed activity of titania-based photocatalysts: Overview and perspective. Catalysts.

[19] Verbruggen S.W. (2015). TiO_2_ photocatalysis for the degradation of pollutants in gas phase: From morphological design to plasmonic enhancement. J. Photoch. Photobio. C.

[20] Kraeutler B., Bard A.J. (1978). Heterogeneous photocatalytic preparation of supported catalysts. Photodeposition of platinum on TiO_2_ powder and other substrates. J. Am. Chem. Soc..

[21] Pichat P., Mozzanega M.N., Disdier J., Herrmann J.M. (1982). Platinum content and temperature effects on the photocatalytic hydrogen production from aliphatic alcohols over platinum/titanium dioxide. Nouv. J. Chim..

[22] Ohtani B., Osaki H., Nishimoto S., Kagiya T. (1986). A novel photocatalytic process of amine N-alkylation by platinized semiconductor particles suspended in alcohols. J. Am. Chem. Soc..

[23] Kowalska E., Remita H., Colbeau-Justin C., Hupka J., Belloni J. (2008). Modification of titanium dioxide with platinum ions and clusters: Application in photocatalysis. J. Phys. Chem. C.

[24] Wei Z., Janczarek M., Wang K., Zheng S., Kowalska E. (2020). Morphology-governed performance of plasmonic photocatalysts. Catalysts.

[25] Zielinska-Jurek A., Klein M., Hupka J. (2017). Enhanced visible light photocatalytic activity of Pt/I-TiO_2_ in a slurry system and supported on glass packing. Sep. Purif. Technol..

[26] Zielinska-Jurek A., Wei Z.S., Janczarek M., Wysocka I., Kowalska E. (2019). Size-controlled synthesis of Pt particles on TiO_2_ surface: Physicochemical characteristic and photocatalytic activity. Catalysts.

[27] Endo-Kimura M., Janczarek M., Bielan Z., Zhang D., Wang K., Markowska-Szczupak A., Kowalska E. (2019). Photocatalytic and antimicrobial properties of Ag_2_O/TiO_2_ heterojunction. ChemEngineering.

[28] Markowska-Szczupak A., Ulfig K., Grzmil B., Morawski A.W. (2010). A preliminary study on antifungal effect of TiO_2_-based paints in natural indoor light. Pol. J. Chem. Technol..

[29] Thabet S., Simonet F., Lemaire M., Guillard C., Cotton P. (2014). Impact of photocatalysis on fungal cells: Depiction of cellular and molecular effects on saccharomyces cerevisiae. Appl. Environ. Microb..

[30] Thabet S., Weiss-Gayet M., Dappozze F., Cotton P., Guillard C. (2013). Photocatalysis on yeast cells: Toward targets and mechanisms. Appl. Catal. B Environ..

[31] Markov S.L., Vidakovic A.M. (2014). Testing methods for antimicrobial activity of TiO2 photocatalyst. Acta Period. Technol..

[32] Kim J.Y., Park C., Yoon J. (2008). Developing a Testing Method for Antimicrobial Efficacy on TiO_2_ Photocatalytic Products. Environ. Eng. Res..

[33] Wang K., Wei Z., Colbeau-Justin C., Nitta A., Kowalska E. (2022). P25 and its components—Electronic properties and photocatalytic activities. Surf. Interfaces.

[34] Wang K.L., Wei Z.S., Ohtani B., Kowalska E. (2018). Interparticle electron transfer in methanol dehydrogenation on platinum-loaded titania particles prepared from P25. Catal. Today.

[35] Ohtani B., Prieto-Mahaney O.O., Li D., Abe R. (2010). What is Degussa (Evonik) P25? Crystalline composition analysis, reconstruction from isolated pure particles and photocatalytic activity test. J. Photoch. Photobiol..

[36] Prieto-Mahaney O.O., Murakami N., Abe R., Ohtani B. (2009). Correlation between photocatalytic activities and structural and physical properties of titanium(IV) oxide powders. Chem. Lett..

[37] Ohno T., Sarukawa K., Tokieda K., Matsumura M. (2001). Morphology of a TiO_2_ photocatalyst (Degussa, P 25) consisting of anatase and rutile crystalline phases. J. Catal..

[38] Gołąbiewska A., Lisowski W., Jarek M., Nowaczyk G., Zielińska-Jurek A., Zaleska A. (2014). Visible light photoactivity of TiO_2_ loaded with monometallic (Au or Pt) and bimetallic (Au/Pt) nanoparticles. Appl. Surf. Sci..

[39] Benz D., Felter K.M., Köser J., Thöming J., Mul G., Grozema F.C., Hintzen H.T., Kreutzer M.T., van Ommen J.R. (2020). Assessing the Role of Pt Clusters on TiO_2_ (P25) on the Photocatalytic Degradation of Acid Blue 9 and Rhodamine B. J. Phys. Chem. C.

[40] Bielan Z., Sulowska A., Dudziak S., Siuzdak K., Ryl J., Zielinska-Jurek A. (2020). Defective TiO_2_ core-shell magnetic photocatalyst modified with plasmonic nanoparticles for visible light-induced photocatalytic activity. Catalysts.

[41] Driessen M.D., Grassian V.H. (1998). Photooxidation of Trichloroethylene on Pt/TiO2. J. Phys. Chem. B.

[42] Paszkiewicz O., Wang K., Rakoczy R., Kordas M., Leniec G., Kowalska E., Markowska-Szczupak A. (2022). Antimicrobial properties of pristine and Pt-modified titania P25 in rotating magnetic field conditions. Chem. Eng. Process. Process Intensif..

[43] Wang K., Kowalska E. (2022). Property-governed performance of platinum-modified titania photocatalysts. Front. Chem..

[44] Lira E., Wendt S., Huo P., Hansen J.Ø., Streber R., Porsgaard S., Wei Y., Bechstein R., Lægsgaard E., Besenbacher F. (2011). The Importance of Bulk Ti^3+^ Defects in the Oxygen Chemistry on Titania Surfaces. J. Am. Chem. Soc..

[45] Wei Z., Endo M., Wang K., Charbit E., Markowska-Szczupak A., Ohtani B., Kowalska E. (2017). Noble metal-modified octahedral anatase titania particles with enhanced activity for decomposition of chemical and microbiological pollutants. Chem. Eng. J..

[46] Baba K., Bulou S., Quesada-Gonzalez M., Bonot S., Collard D., Boscher N.D., Choquet P. (2017). Significance of a noble metal nanolayer on the UV and visible light photocatalytic activity of anatase TiO_2_ thin films grown from a scalable PECVD/PVD approach. ACS Appl. Mater. Inter..

[47] Li Y., Wang C., Zhang C., He H. (2020). Formaldehyde Oxidation on Pd/TiO_2_ Catalysts at Room Temperature: The Effects of Surface Oxygen Vacancies. Top. Catal..

[48] Murcia J.J., Hidalgo M.C., Navío J.A., Vaiano V., Ciambelli P., Sannino D. (2012). Photocatalytic Ethanol Oxidative Dehydrogenation over Pt/TiO_2_: Effect of the Addition of Blue Phosphors. Int. J. Photoenergy.

[49] Song L., Lu Z., Zhang Y., Su Q., Li L. (2018). Hydrogen-Etched TiO_2−x_ as Efficient Support of Gold Catalysts for Water–Gas Shift Reaction. Catalysts.

[50] Abdelouahab Reddam H., Elmail R., Lloria S.C., Monrós Tomás G., Reddam Z.A., Coloma-Pascual F. (2020). Synthesis of Fe, Mn and Cu modified TiO_2_ photocatalysts for photodegradation of Orange II. Bol. Soc. Esp. Ceram. Vidr..

[51] Shu Z., Cai Y., Ji J., Tang C., Yu S., Zou W., Dong L. (2020). Pt Deposites on TiO_2_ for Photocatalytic H_2_ Evolution: Pt Is Not Only the Cocatalyst, but Also the Defect Repair Agent. Catalysts.

[52] Siuzdak K., Sawczak M., Klein M., Nowaczyk G., Jurga S., Cenian A. (2014). Preparation of platinum modified titanium dioxide nanoparticles with the use of laser ablation in water. Phys. Chem. Chem. Phys..

[53] Liu S., Qi H., Zhou J., Xu W., Niu Y., Zhang B., Zhao Y., Liu W., Ao Z., Kuang Z. (2021). Encapsulation of Platinum by Titania under an Oxidative Atmosphere: Contrary to Classical Strong Metal–Support Interactions. ACS. Catal..

[54] Endo M., Wei Z.S., Wang K.L., Karabiyik B., Yoshiiri K., Rokicka P., Ohtani B., Markowska-Szczupak A., Kowalska E. (2018). Noble metal-modified titania with visible-light activity for the decomposition of microorganisms. Beilstein J. Nanotech..

[55] Sabino R., Veríssimo C., Viegas C., Viegas S., Brandão J., Alves-Correia M., Borrego L.-M., Clemons K.V., Stevens D.A., Richardson M. (2019). The role of occupational Aspergillus exposure in the development of diseases. Med. Mycol..

[56] Abdel Hameed A.A., Ayesh A.M., Abdel Razik Mohamed M., Abdel Mawla H.F. (2012). Fungi and some mycotoxins producing species in the air of soybean and cotton mills: A case study. Atmos. Pollut. Res..

[57] Noman E., Al-Gheethi A., Saphira Radin Mohamed R.M., Talip B., Othman N., Hossain S., Vo D.-V.N., Alduais N. (2022). Inactivation of fungal spores from clinical environment by silver bio-nanoparticles; optimization, artificial neural network model and mechanism. Environ. Res..

[58] Godlewska-Żyłkiewicz B., Sawicka S., Karpińska J. (2019). Removal of Platinum and Palladium from Wastewater by Means of Biosorption on Fungi Aspergillus sp. and Yeast Saccharomyces sp.. Water.

[59] Mukherjee K., Acharya K., Biswas A., Jana N.R. (2020). TiO_2_ Nanoparticles Co-doped with Nitrogen and Fluorine as Visible-Light-Activated Antifungal Agents. ACS Appl. Nano Mater..

[60] Mendez-Medrano M.G., Kowalska E., Endo M., Wang K., Bahena D., Rodriguez-Lopez J.L., Remita H. (2019). Inhibition of fungal growth using modified TiO_2_ with core@shell structure of Ag@CuO clusters. ACS Appl. Bio Mater..

[61] Krishnan A., Convey P., Gonzalez-Rocha G., Alias S.A. (2016). Production of extracellular hydrolase enzymes by fungi from King George Island. Polar Biol..

[62] Calado T., Venâncio A., Abrunhosa L. (2014). Irradiation for Mold and Mycotoxin Control: A Review. Compr. Rev. Food Sci. Food Saf..

[63] Yagyu Y., Sakudo A., Shintani H., Sakudo A. (2016). Current technology and applications of gas plasma for disinfection of agricultural products: Disinfection of fungal spores on *Citrus unshiu* by atmospheric pressure dielectric barrier discharge. Gas Plasma Sterilization in Microbiology: Theory, Applications, Pitfalls and New Perspectives.

[64] Ji C., Fan Y., Zhao L. (2016). Review on biological degradation of mycotoxins. Anim. Nutr..

[65] Rogawansamy S., Gaskin S., Taylor M., Pisaniello D. (2015). An Evaluation of Antifungal Agents for the Treatment of Fungal Contamination in Indoor Air Environments. Int. J. Env. Res. Pub. Health.

[66] Hojnik N., Modic M., Ni Y., Filipič G., Cvelbar U., Walsh J.L. (2019). Effective Fungal Spore Inactivation with an Environmentally Friendly Approach Based on Atmospheric Pressure Air Plasma. Environ. Sci. Technol..

[67] Escudero-Leyva E., Alfaro-Vargas P., Muñoz-Arrieta R., Charpentier-Alfaro C., Granados-Montero M.d.M., Valverde-Madrigal K.S., Pérez-Villanueva M., Méndez-Rivera M., Rodríguez-Rodríguez C.E., Chaverri P. (2022). Tolerance and Biological Removal of Fungicides by Trichoderma Species Isolated From the Endosphere of Wild Rubiaceae Plants. Front. Agron..

